# Relationship Between Food Selectivity, Adaptive Functioning and Behavioral Profile in Individuals with Autism Spectrum Disorder

**DOI:** 10.3390/bs15121664

**Published:** 2025-12-02

**Authors:** Rachele Sarnataro, Martina Siracusano, Roberta Campanile, Claudia Marcovecchio, Silvia Babolin, Assia Riccioni, Lucrezia Arturi, Luigi Mazzone

**Affiliations:** 1 Child Neurology and Psychiatry Unit, Department of Wellbeing of Mental and Neurological, Dental and Sensory Organ Health, Policlinico Tor Vergata Hospital, 00133 Rome, Italy; rachelesarnataro97@gmail.com (R.S.); roby.campanile.92@gmail.com (R.C.); silviababolin@hotmail.com (S.B.); assiariccioni@gmail.com (A.R.); luigi.mazzone@uniroma2.it (L.M.); 2 Department of Biomedicine and Prevention, University of Rome Tor Vergata, 00133 Rome, Italy; 3 Systems Medicine Department, University of Rome Tor Vergata, 00133 Rome, Italy; claudia.marcovecchio3@gmail.com (C.M.); lucrezia.arturi@gmail.com (L.A.)

**Keywords:** food selectivity, sensory processing, autism, behavioral profile, adaptive skills

## Abstract

Objectives: Atypical eating habits frequently characterize people with Autism Spectrum Disorder (ASD) from early infancy. Food selectivity, defined as a narrow variety of food intake and reticence to new food, is the most frequent feeding disorder in ASD. The objective of this study was to investigate the adaptive functioning and the behavioral profile of individuals with ASD with food selectivity (FS) in comparison to an ASD sample without food selectivity (NFS). Methods: We conducted a retrospective study on 286 children (mean age = 46.95 months) with a diagnosis of ASD; 43.3% of the sample had a history of food selectivity (FS), whereas 56.6% had no history of food selectivity (NFS). Results: No differences were found between the FS and NFS groups on cognition, autism symptom levels, and age. The FS group presented lower adaptive skills and greater behavioral problems in comparison to the NFS group. A worse clinical profile characterized children with more than one kind of food selectivity. Conclusions: The early identification and longitudinal evaluation of specific clinical and behavioral patterns in children with ASD associated with food selectivity could contribute to a better understanding of the relationship between autism symptoms and atypical eating habits.

## 1. Introduction

Autism Spectrum Disorder (ASD) is a neurodevelopmental condition—defined as a spectrum condition due to the broad variability in symptoms—that may manifest during the first years of life. However, depending on the severity of symptoms, a diagnosis may not be clear until school age. It is characterized by impairments in two core domains: social communication and reciprocity, as well as restricted and repetitive patterns of behavior, interest, and activities (American Psychiatric Association, 2013). Both of these features must be present at a young age and be severe enough to significantly impair the child’s ability to function at home, school, or other situations.

Children with ASD usually exhibit atypical eye contact; difficulty interpreting others’ body language, gestures, and expressions; and diminished facial expressions, and they may fail to initiate or respond to social interactions or conversation. Moreover, they show stereotyped or repetitive language or movements, inflexible adherence to routines and rituals, highly restricted interests, and extreme under- or overreaction to external stimuli.

Individuals with ASD can experience speech delay and/or an intellectual disability; in fact, these two characteristics are included as two specifiers in the DSM-5 (American Psychiatric Association, 2013) due to their high prevalence in ASD. Actually, rates of co-morbid intellectual disability (ID) in patients with ASD are reported at 31.0% (26.7% to 39.4%) with ID defined as an intelligence quotient (IQ) ≤ 70 (Hodges et al., 2019). Speech is typically delayed or may regress in almost a third of patients with ASD: nonverbal and verbal language are impaired, and some children are nonverbal or have strongly limited speech abilities (Rapin & Tuchman, 2008).

In addition to the core symptoms, ASD is often associated with a range of co-occurring medical and psychiatric issues, including seizures and epilepsy (possibly due to the common pathophysiological mechanisms that account for both the autism and the epilepsy) (Tuchman & Rapin, 2002), impaired sleep (with a prevalence ranging from 40% to 86%), and immune and gastrointestinal disorders (Rossignol & Frye, 2011; Gesundheit et al., 2013; Buie et al., 2010).

Although not a diagnostic feature of ASD, feeding problems are common in this clinical population (between 46% and 89%) and food selectivity is the most frequent one (Nadon et al., 2011; Ledford & Gast, 2006).

The term “food selectivity” refers to a heterogenous constellation of feeding-related behaviors, including the rejection of specific foods, flavors, colors, textures, temperatures, food packaging, and presentation. It may also manifest as adherence to highly restricted dietary repertoires limited to specific food categories, reflecting pronounced rigidity in food preferences and a reduced acceptance of new foods. Food selectivity is frequently associated with dysfunctional mealtime behaviors such as crying, yelling, running away, aggression, spitting, food throwing, and prolonged chewing without swallowing. These ritualized and repetitive behaviors contribute to the characterization of food selectivity.

When examining feeding issues in ASD, both medical or sensorial/behavioral characteristics have been identified as contributors to food refusal or restricted food preferences of autistic children during mealtimes (Esposito et al., 2023). Several studies have observed that gastrointestinal problems might be linked to pronounced food selectivity in some of these individuals (Ibrahim et al., 2009). Furthermore, it has been suggested that sensory processing problems could be underlying mechanisms of food selectivity in this population (Suarez et al., 2014; Mirizzi et al., 2025).

Postorino et al. (2015) reported that children exhibiting food selectivity showed higher levels of ASD symptoms on parent-reported questionnaires, whereas this association was not detected using clinician-rated measures. This discrepancy may reflect different perspectives between parents and clinicians: parents who are directly engaged in mealtime management may encounter more severe behavioral challenges and greater functional impairment in children with ASD and food selectivity.

A recent meta-analysis by Ferrara et al. (2025) further confirmed that children diagnosed with ASD show an increased prevalence of feeding problems and food selectivity, which can lead to clinically relevant nutritional deficiencies. Moreover, it emphasizes the significant role of sensory processing disturbances in food selectivity and maladaptive and disruptive mealtime behaviors. These findings are consistent with previous evidence, including the comprehensive meta-analysis by Margari et al. (2020), which found limited consensus regarding the association between cognitive level, adaptive skills, ASD symptoms, and food selectivity. Nevertheless, the authors confirmed a substantial link between sensory hypersensitivity and food selectivity, and highlighted that behavioral problems, particularly repetitive behaviors and internalizing/externalizing symptoms, tend to be more prevalent among children with feeding difficulties.

Despite the growing body of literature, to the best of our knowledge, there are no studies characterizing the influence of different types of food selectivity on specific clinical profiles in children with ASD. The present study primarily aims to investigate the adaptive functioning and behavioral profile in ASD individuals with food selectivity, with particular focus on differentiating subtypes of food selectivity. Comparisons will be made with a cohort of ASD individuals without food selectivity, in order to determine whether selective eating behaviors are associated with variations in externalizing and internalizing symptoms, core social and communication deficits, or cognitive functioning.

## 2. Materials and Methods

### 2.1. Study Design and Enrollment of Participants

For the purpose of this retrospective study, we analyzed the available electronical health records (anamnestic information, cognitive, behavioral, and adaptive functioning data, as well as feeding-related information) of children diagnosed with ASD who were undergoing clinical follow-up at the Child Neuropsychiatry Unit of Rome Tor Vergata University-Hospital (Italy). The final sample included 286 children with ASD, aged between 1 month and 5 years (age M: 46.95 months ± DS 11.02), of whom 229 were males (80.1%) and 57 were females (19.9%).

Exclusion criteria included the presence of a known genetic syndrome or comorbid epilepsy.

### 2.2. Clinical Data

In our retrospective study, we used information and data previously collected and included on our database.

#### 2.2.1. Cognitive Skills

Griffiths Mental Developmental Scale—Third Edition (GMDS-III), Leiter-Revised (Leiter-R), or Wechsler Preschool and Primary Scale of Intelligence—Third Edition (WPPSI-III) (Green et al., 2016; Roid & Miller, 1997; Wechsler, 2002) have been considered, depending upon children’s age.

GMDS-III (from birth to 6 years of age) comprises five scales that are expressed as quotients to provide a General Development Quotient (GDQ).

Leiter-R (2–20 years of age) is a culture-free test that measures non-verbal IQ and is especially useful for kids and teenagers with verbal impairment and/or cognitive delay.

WPPSI-III (from 2 years and 6 months to 7 years and 3 months) provides a Total IQ, Verbal IQ (VIQ), performance IQ (PIQ), Processing Speed Quotient (PSQ), and a Total general language score (GL).

#### 2.2.2. Adaptive Functioning

The Adaptive Behavior Assessment System-second edition, 0–5 years, Italian edition (Oakland, 2011), has been considered for the evaluation of children’s everyday adaptive skills. The items are rated on a Likert scale ranging from 0 to 3 (0 = Not able to do; 1 = Able to do and never performs; 2 = Able to do and sometimes performs; 3 = Able to do and always performs) and are grouped into 10 skill areas, combined to provide three composite scores: conceptual adaptive domain (DAC) (communication, preschool skills, self-control), social adaptive domain (DAS) (play and social skills), and practical adaptive domain (DAP) (self-care, life at home/school, use of the environment, health and safety, work). These three domains are unified in a total score, the General Adaptive Composite (GAC).

#### 2.2.3. ASD Symptoms Severity

The Autism Diagnostic Observation Schedule Second Edition (ADOS-2) (Lord et al., 2012) has been employed to define a clinical ASD diagnosis. The ADOS-2′s calibrated severity score (CSS), ranging from 0 to 10, was used to measure the severity of the autistic symptoms (Hus et al., 2014; Gotham et al., 2009). To make it easier to compare the Toddler Module of ADOS-2 directly to other modules, the total CSS was determined based on Esler et al. (2015).

#### 2.2.4. Behavioral Profile

Parents’ Child Behaviour Checklist ½-5 (CBCL) (Achenbach & Rescorla, 2000), Italian version (Frigerio et al., 2006), results have been employed to evaluate children’s behavioral problems. Each item is rated on a Likert scale ranging from 0 to 2 (0 = Not true; 1 = Sometimes true; 2 = Very often true). By converting the raw scores into T scores, the checklist produces eight syndromic scales that contribute to two general dimensions (internalizing and externalizing problems) and one total score.

#### 2.2.5. Food Selectivity

Information about food selectivity was obtained from anamnestic data available in our clinical database for ASD patients. During routine clinical assessments, parents were asked to provide a detailed description of their child’s eating habits, with particular attention to sensory and food-related factors associated with three predefined categories of food selectivity: “texture”, “color and/or presentation”, and “both selectivity”. To characterize these patterns, parents were asked some exploratory questions, aimed at identifying specific food characteristics associated with refusal. Examples of these questions included the following:

“Does your child eat all the foods you prepare for them?”

“Does your child refuse foods based on their color? For example, do they accept green, yellow or red food?”

“Does your child refuse food based on their texture, for instance, crunchy, creamy or hard foods?”

“Does your child accept different types of foods presented together on the same plate?”

These parent-reported descriptions were subsequently categorized to determine the presence and type of food selectivity for each child.

### 2.3. Data Analyses

Data analyses were conducted using the Statistical Package for Social Sciences (SPSS Statistic 25 for Windows). Mann–Whitney analyses were employed for dichotomous variables, and the Kruskal–Wallis test was applied to continuous variables. Non-parametric tests were selected because the distribution of the variables deviated from normality, as determined by the Shapiro–Wilk test (*p* > 0.05). Given the presence of several outliers and heterogeneous distributions across groups for certain measures, the k-sample median test (also referred to as Mood’s median test) was additionally performed. Effect size in statistically significant results was calculated for post hoc pairwise comparisons made with the Mann–Whitney test, using the formula R = Z/√N. Furthermore, since cognitive functioning was categorized into two levels (< or >70), chi-square analyses were conducted to examine potential associations between cognitive level and type of food selectivity.

An alpha level of 0.05 was set for statistical significance.

## 3. Results

### 3.1. Participants’ Demographical and Food Selectivity Profiles

The study sample consisted of 286 children with ASD, aged between 1 month and 5 years (M = 46.95 months ± DS 11.02), of whom 229 were males (80.1%) and 57 were females (19.9%). Regarding cognitive functioning, 116 children demonstrated age-appropriate cognitive skills (40.6%), whereas 170 (59.4%) met the criteria for comorbid intellectual disability (ID).

Regarding feeding problems, 124 children (43.3%, 98 males and 26 females) had a parent-reported history of food selectivity (FS), while 162 (56.6%, 131 males and 31 females) had no history of food selectivity (NFS). The two groups were matched for age and sex.

Within the FS group, 35 children (12.2%) exhibited selectivity based on food color and/or presentation, whereas 27 (9.4%) demonstrated both types of selectivity.

### 3.2. Participants’ Clinical Symptoms, Adaptive Skills, and Behavioral Profile

Autistic symptoms, assessed using the ADOS severity score (CSS), showed no significant differences between FS and NFS groups according to the Mann–Whitney test. This result was also confirmed by Kruskal–Wallis analysis, which also found no differences in ADOS-2 CSS between children with specific subtypes of food selectivity and those without food selectivity.

Cognitive abilities were assessed according to children’s age using Leiter-R (n = 88), WPPSI-II (n = 10) or GMDS-II (n = 188). No significant differences emerged in cognitive/developmental profile between FS and NFS groups, whether comparing the two groups directly (with the Mann–Whitney test) or across subtypes of FS (with the Kruskal–Wallis test). Similarly, no significant differences were found, with both analyses, in children’s median age.

**Figure 1:**
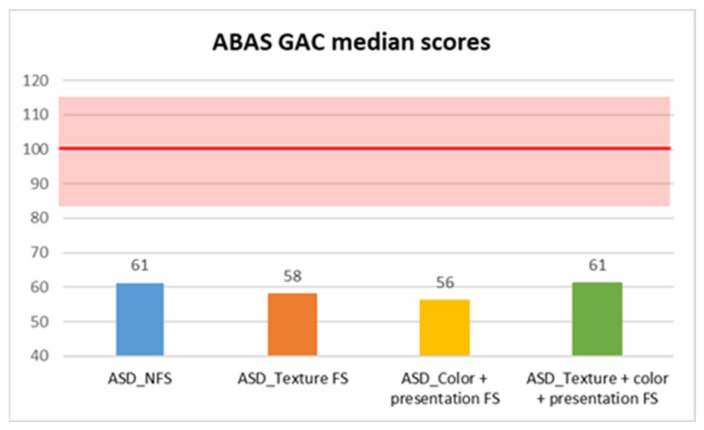
ABAS GAC median scores in autistic individuals with and without food selectivity and different types of food selectivity. Legend: Adaptive Behavior Assessment System-second edition (ABAS); General Adaptive Composite (GAC); Autism Spectrum Disorder (ASD); no food selectivity (NFS); food selectivity for texture (Texture FS); food selectivity for color and presentation (Color + Presentation FS); food selectivity for texture, color, and presentation (Texture + Color + Presentation FS). Red highlighted area: normal results area (100 ± 15).

**Figure 2:**
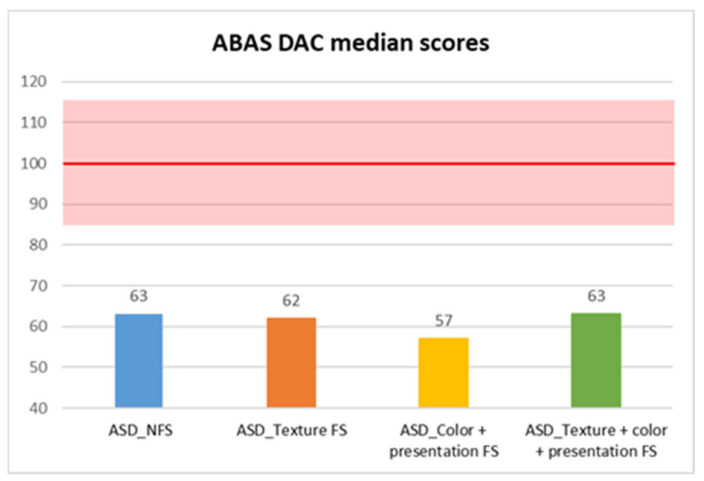
ABAS DAC median scores in autistic individuals with and without food selectivity and different types of food selectivity. Legend: Adaptive Behavior Assessment System-second edition (ABAS); conceptual adaptive domain (DAC); Autism Spectrum Disorder (ASD); no food selectivity (NFS); food selectivity for texture (Texture FS); food selectivity for color and presentation (Color + Presentation FS); food selectivity for texture, color, and presentation (Texture + Color + Presentation FS). Red highlighted area: normal results area (100 ± 15).

**Figure 3:**
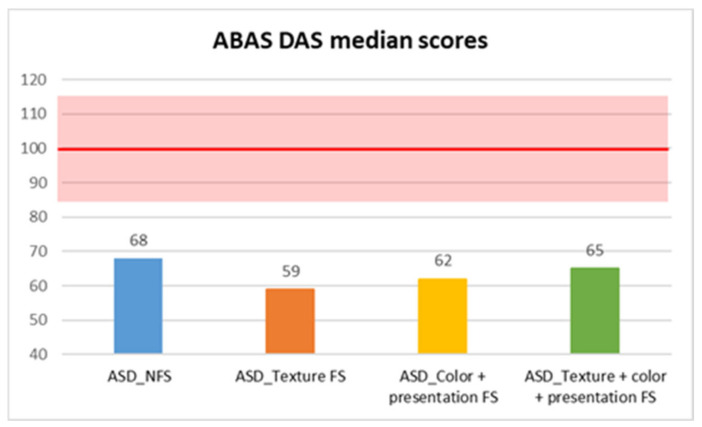
ABAS DAS median scores in autistic individuals with and without food selectivity and different types of food selectivity. Legend: Adaptive Behavior Assessment System-second edition (ABAS); social adaptive domain (DAS); Autism Spectrum Disorder (ASD); no food selectivity (NFS); food selectivity for texture (Texture FS); food selectivity for color and presentation (Color + Presentation FS); food selectivity for texture, color, and presentation (Texture + Color + Presentation FS). Red highlighted area: normal results area (100 ± 15).

**Figure 4:**
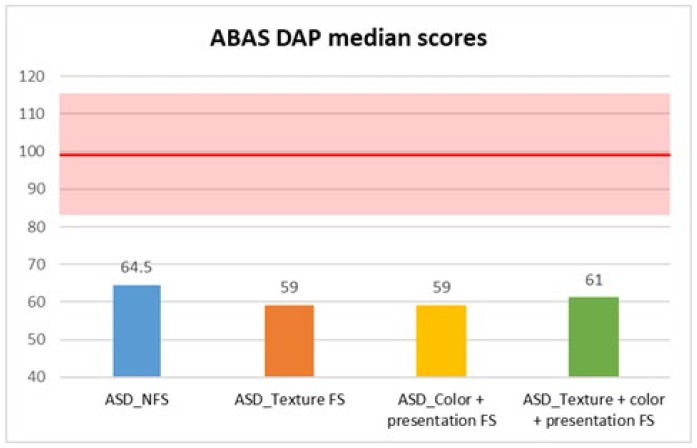
ABAS DAP median scores in autistic individuals with and without food selectivity and different types of food selectivity. Legend: Adaptive Behavior Assessment System-second edition (ABAS); practical adaptive domain (DAP); Autism Spectrum Disorder (ASD); no food selectivity (NFS); food selectivity for texture (Texture FS); food selectivity for color and presentation (Color + Presentation FS); food selectivity for texture, color, and presentation (Texture + Color + Presentation FS). Red highlighted area: normal results area (100 ± 15).

In contrast, adaptive skills assessed through the parent-reported ABAS questionnaire showed statistically significant differences between FS and NFS groups on ABAS GAC total score (*p* = 0.004, R = 0.17), as well as in DAC (*p* = 0.042, R = 0.12), DAS (*p* = 0.002, R = 0.18), and DAP (*p* < 0.001, R = 0.21) subscales. In more detail, median scores were consistently lower in the FS group in GAC (median: 57 ± 14.59), DAC (median: 61 ± 17.54), DAS (median: 62 ± 14.71), and DAP (median: 59 ± 14.5) subscales, compared with the NFS group (GAC median: 61±14.72; DAC median: 63 ± 15.86; DAS median: 68 ± 13.83; DAP median: 64.5 ± 16.17) (Figure 1, Figure 2, Figure 3 and Figure 4).

Moreover, we also compared different types of food selectivity with a median test (since there was no equal distribution between different groups, so we could not use Kruskal–Wallis’ test), and we observed statistically significant differences in two domains of adaptive skills: in DAS scores between children with NFS (median: 68 ± 13.83) and children with food texture selectivity (median: 59 ± 14.48) with a *p* value of 0.004, and in DAP scores between children with texture selectivity (median: 59 ± 14.88) or both selectivity (median: 61 ± 10.12) and children with NFS (median: 64.5 ± 16.18), in the former case with a *p* value of 0.002 and in the latter with a *p* value of 0.02 (Table 1, Table 2 and Table 3).

**Figure 5:**
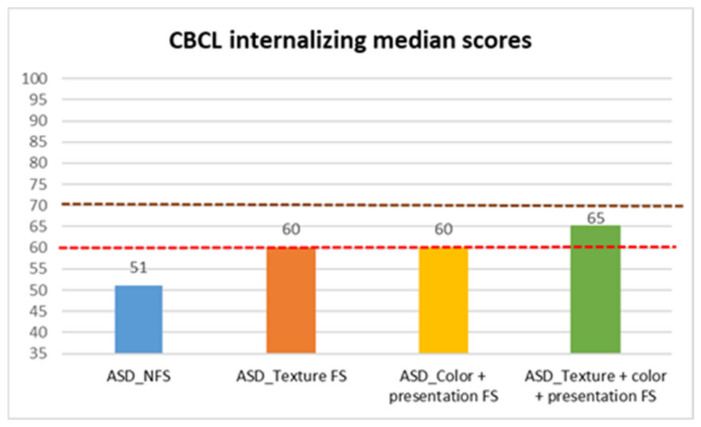
CBCL internalizing median scores in autistic individuals with and without food selectivity and different types of food selectivity. Legend: Child Behavior Checklist (CBCL); Autism Spectrum Disorder (ASD); no food selectivity (NFS); food selectivity for texture (Texture FS); food selectivity for color and presentation (Color + Presentation FS); food selectivity for texture color and presentation (Texture + Color + Presentation FS). Red dotted line: borderline scores. Burgundy dotted line: significant scores.

**Figure 6:**
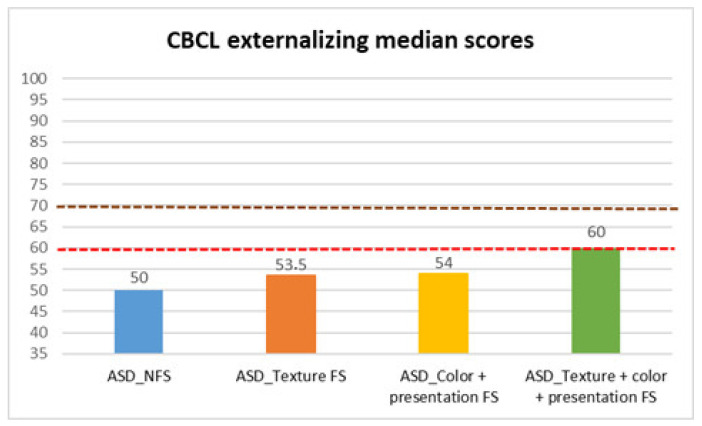
CBCL externalizing median scores in autistic individuals with and without food selectivity and different types of food selectivity. Legend: Child Behavior Checklist (CBCL); Autism Spectrum Disorder (ASD); no food selectivity (NFS); food selectivity for texture (Texture FS); food selectivity for color and presentation (Color + Presentation FS); food selectivity for texture, color, and presentation (Texture + Color + Presentation FS). Red dotted line: borderline scores. Burgundy dotted line: significant scores.

**Figure 7:**
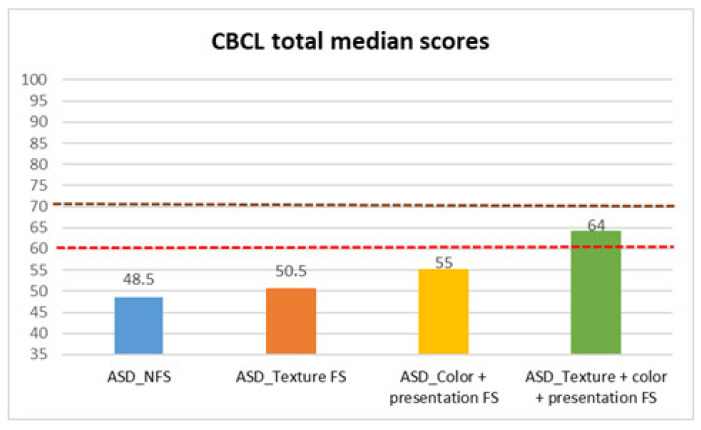
CBCL total median scores in autistic individuals with and without food selectivity and different types of food selectivity. Legend: Child Behavior Checklist (CBCL); Autism Spectrum Disorder (ASD); no food selectivity (NFS); food selectivity for texture (Texture FS); food selectivity for color and presentation (Color + Presentation FS); food selectivity for texture, color, and presentation (Texture + Color + Presentation FS). Red dotted line: borderline scores. Burgundy dotted line: significant scores.

Finally, we analyzed CBCL scores (Table 4, Table 5 and Table 6, Figure 5, Figure 6 and Figure 7), to evaluate children’s behavioral problems. With the Mann–Whitney test, we found a statistically significant difference between FS and NFS groups on the CBCL total scale (*p* < 0.001, R = 0.23), as well as internalizing (*p* < 0.001, R = 0.32) and externalizing (*p* < 0.001, R = 0.23) CBCL subscales. In more detail, parents of the FS group reported a larger degree of impairment on the CBCL total (median: 55 ± 10.38), internalizing (median: 61 ± 9.31), and externalizing (median: 54.5 ± 8.54) scores, compared to parents of the NFS group (CBCL total median: 48.5 ± 8.93; CBCL internalizing median: 51 ± 9.88; CBCL externalizing median: 50 ± 9.11).

Median test analyses across FS subtypes also revealed significant differences.

In CBCL total scores, parents of children with both selectivity (median: 64 ± 9.71) and color and/or presentation (median: 55 ± 9.94) reported greater behavioral problems compared to parents of children with NFS (median: 48.5 ± 8.93), in the latter case with a p value of 0.004 and in the former with a p value of 0.022. Children with both types of food selectivity also showed statistically significant higher scores (median: 64 ± 9.71) than those with texture selectivity (median: 50.5 ± 10.33) (*p* = 0.009) and those with presentation and/or color selectivity (median: 55 ± 9.94) (*p* = 0.025).

In CBCL internalizing scores, parents of children with both selectivity (median: 65 ± 7.37, *p* < 0.001), color and/or presentation (median: 60 ± 9.87, *p* = 0.041), and texture selectivity (median: 60 ± 9.46, *p* ≤ 0.001) described greater behavioral problems compared to parents of children with NFS (median: 51 ± 9.88). Additionally, children with both types of selectivity scored significantly higher than those with texture (*p* = 0.035) and color/presentation (*p* = 0.041) selectivity.

In CBCL externalizing scores, parents of children with both selectivity (median: 60 ± 8.47) described more problems, compared to parents of children with NFS (median: 50 ± 9.11), with a p value of 0.001.

Multiple regression analyses were performed to evaluate the effects of potential confounders. In the ABAS subscales (GAC, DAC, DAS, DAP), cognitive and behavioral variables such as gender, age, IQ (average or lower than 70), and CSS significantly influenced scores, whereas the type of food selectivity had only a partial effect. Specifically, for the GAC scale, the overall model explains approximately 17.6% of the variance (R^2^ *=* 0.176; *p* < 0.001), with significant effects of age (β = −0.277; *p* < 0.001) and IQ (β = −0.264; *p* < 0.001). Similar results were observed for the other subscales—DAC (R^2^ = 0.164; *p* < 0.001), DAS (R^2^ = 0.187; *p* < 0.001), and DAP (R^2^ = 0.143; *p* < 0.001)—where the same predictors showed significant negative effects.

Regarding the CBCL, the type of food selectivity was positively associated with behavioral scores: CBCL_int (β = 0.307; *p* < 0.001), CBCL_est (β = 0.243; *p* < 0.001), and CBCL_tot (β = 0.292; *p* < 0.001), with explained variances of 12.6%, 6.5%, and 10%, respectively (Table 7).

## 4. Discussion

A substantial body of research has shown that food selectivity represents one of the most frequent behavioral challenges in children with ASD (Nadon et al., 2011; Bandini et al., 2010; Schmitt et al., 2008; Dominick et al., 2007). Reported prevalence rates of food selectivity among individuals with ASD vary widely, ranging from 13% to 87% (Nadon et al., 2011; Bandini et al., 2010; Schmitt et al., 2008; Dominick et al., 2007).

To further characterize potential distinctive features associated with food selectivity in the ASD population, we examined behavioral and clinical differences between ASD children with and without food selectivity. Our findings revealed no significant group differences between groups on cognition, autism symptom severity, and age. Conversely, clear differences emerged in adaptive functioning and general behavior, with parents of children with FS reporting lower adaptive skills and greater behavioral problems compared to parents of children with NFS. More specifically, children with texture-based selectivity showed lower social and practical adaptive skills; children with both texture and color–presentation selectivity demonstrated reduced practical adaptive skills and higher internalizing, externalizing, and total behavioral problems.

Our results suggest that ASD children with food selectivity exhibit more behavioral difficulties and fewer adaptive skills than their non-selective counterparts, based on parent-reported questionnaires (i.e., ABAS and CBCL).

Multivariate analysis further suggests that higher levels of food selectivity are associated with greater behavioral difficulties—particularly internalizing symptoms—while adaptive functioning tends to decrease as cognitive impairment and autism symptoms increase. 

Furthermore, parents of children with more than one type of food selectivity reported significantly higher levels of their children’s behavioral problems and lower scores in specific adaptive skills domains, such as the social and practical ones.

One possible explanation for these findings may lie in parental perception: parents of children with ASD and food selectivity may perceive their child to have significantly more pronounced behavioral, social, and practical difficulties compared to parents of children with ASD without food selectivity, with this perception being even stronger when multiple forms of selectivity are present. 

Our results are consistent with previous studies indicating that ASD children with food selectivity tend to show more challenging behavioral and mealtime problems and adaptive profiles (Chen et al., 2022; Postorino et al., 2015; Wenzell et al., 2024; Li et al., 2025; Curtin et al., 2015). Notably, these studies originate from various countries, including Italy, the United States, and Japan, suggesting that difficulties associated with food selectivity may transcend cultural and geographic contexts. 

Future research would benefit from examining potential cross-cultural differences in feeding behaviors, given the variability in mealtime routines and dietary habits across countries. Indeed, many existing questionnaires that assess children’s food preferences—such as the Food Frequency Questionnaire and the Diet History Questionnaire—are based on American dietary patterns and may be less suitable for investigating habits in Italy or other cultural contexts. 

Although this study enriches the existing literature concerning food selectivity in children with ASD, several limitations should be acknowledged. The main limitations of the study are represented by the lack of a typically developing control group and the absence of a standardized measure of food selectivity. In fact, food habits were not derived from a daily food diary, nor from standardized interviews or questionnaires, but only from parental verbal reports.

Finally, as a cross-sectional study, the findings are valuable for generating hypotheses, but require confirmation through future multicentric longitudinal research, incorporating standardized measures of food selectivity, including an evaluation of multisensory processing and parameters of nutritional status.

## 5. Conclusions

In conclusion, while food selectivity has been extensively examined in ASD populations, further research is needed to deepen our understanding of its clinical phenomenology.

Identifying specific clinical and behavioral patterns in children with ASD and food selectivity is crucial for parents and clinicians, as such knowledge can inform daily management and guide the development of targeted interventions and treatment strategies.

## Figures and Tables

**Table 1:** Social and practical adaptive behavior scores and standard deviation in autistic individuals with and without food selectivity.

**ABAS** **DAS**	**ASD_NFS**	**Median**	**68.00**
		**Standard Deviation**	**13.834**
	**ASD_FS**		
	Texture FS	Median	59.00
		Standard deviation	14.479
	Color + presentation FS	Median	62.00
		Standard deviation	17.428
	Texture + color + presentation FS	Median	65.00
		Standard deviation	11.222
**ABAS** **DAP**	**ASD_NFS**	Median	64.50
		Standard deviation	16.179
	**ASD_FS**		
	Texture FS	Median	59.00
		Standard deviation	14.881
	Color + presentation FS	Median	59.00
		Standard deviation	16.793
	Texture + color + presentation FS	Median	61.00
		Standard deviation	10.116

Legend: Adaptive Behavior Assessment System-second edition (ABAS); Autism Spectrum Disorder (ASD); food selectivity (FS); no food selectivity (NFS); social adaptive domain (DAS); practical adaptive domain (DAP).

**Table 2:** Mann–Whitney comparisons of ABAS subscales between FS and NFS.

Subscale	FS (Median)	NFS (Median)	U	Z	*p*-Value	Effect Size
**GAC**	61	57	8070.5	−2.85	0.004 **	0.17
**DAC**	63	61	8634.5	−2.04	0.042 *	0.12
**DAS**	68	62	7942.5	−3.04	0.002 **	0.18
**DAP**	64.5	59	7566.0	−3.58	<0.001 ***	0.21

Legend: Adaptive Behavior Assessment System-second edition (ABAS); General Adaptive Composite (GAC); conceptual adaptive domain (DAC); social adaptive domain (DAS); practical adaptive domain (DAP); food selectivity (FS); no food selectivity (NFS). Note: U = Mann–Whitney U statistic; Z = standardized test statistic; *p*-value * = *p* < 0.05, ** = *p* < 0.01, *** = *p* < 0.001.

**Table 3:** Median test and post hoc pairwise comparisons of ABAS across different types of food selectivity.

Subscale	χ^2^	df	*p*-Value	Post Hoc Pairwise Comparisons	Post Hoc *p*-Value	Effect Size
**GAC**	5.343	3	0.148	—		
**DAC**	1.123	3	0.772	—		
**DAS**	8.265	3	0.041	Color + Presentation FS vs. NFS	0.004 **	0.480
**DAP**	10.770	3	0.013	Color + Presentation FS vs. NFS	0.002 **	0.566
				All_FS vs. NFS	0.020 **	0.319

Legend: Adaptive Behavior Assessment System-second edition (ABAS); General Adaptive Composite (GAC); conceptual adaptive domain (DAC); social adaptive domain (DAS); practical adaptive domain (DAP); food selectivity (FS); no food selectivity (NFS); Color + Presentation + Texture FS (All_FS). Note: χ^2^ = chi-square statistic; df = degrees of freedom. *p*-value ** = *p* < 0.01.

**Table 4:** CBCL median scores and standard deviation of autistic individuals with and without food selectivity.

**CBCL internalizing**	**ASD_NFS**	**Median**	**51.00**
		**Standard Deviation**	**9.879**
	**ASD_FS**		
	Texture FS	Median	60.00
		Standard deviation	9.464
	Color + presentation FS	Median	60.00
		Standard deviation	9.869
	Texture + color + presentation FS	Median	65.00
		Standard deviation	7.369
**CBCL externalizing**	**ASD_NFS**	Median	50.00
		Standard deviation	9.109
	**ASD_FS**		
	Texture FS	Median	53.50
		Standard deviation	8.706
	Color + presentation FS	Median	54.00
		Standard deviation	7.829
	Texture + color + presentation FS	Median	60.00
		Standard deviation	8.469
**CBCL total**	**ASD_NFS**	Median	48.50
		Standard deviation	8.935
	**ASD_FS**		
	Texture FS	Median	50.50
		Standard deviation	10.333
	Color + presentation FS	Median	55.00
		Standard deviation	9.94
	Texture + color + presentation FS	Median	64.00
		Standard deviation	9.715

Legend: Child Behavior Checklist (CBCL); Autism Spectrum Disorder (ASD); food selectivity (FS); no food selectivity (NFS).

**Table 5:** Mann–Whitney comparisons of CBCL subscales between FS and NFS.

Subscale	NFS (Median)	FS (Median)	U	Z	*p*	Effect Size
Internalizing	51	61	13,758.5	5.37	<0.001 ***	0.23
Externalizing	50	54.5	12,714.5	3.86	<0.001 ***	0.32
Total	48.5	55	12,782.0	3.95	<0.001 ***	0.23

Legend: Child Behavior Checklist (CBCL); food selectivity (FS); no food selectivity (NFS). Note: U = Mann–Whitney U statistic; Z = standardized test statistic; *p*-value *** = *p* < 0.001.

**Table 6:** Median test and post hoc pairwise comparisons of CBCL across different types of food selectivity.

Subscale	χ^2^	df	*p*	Post Hoc Pairwise Comparisons	*p*-Value	Effect Size
**CBCL Internalizing**	31.947	3	<0.001	Texture FS vs. All FS	0.035 *	0.263
				Color + Presentation vs. All FS	0.044 *	0.241
				NFS vs. Texture FS	<0.001 ***	0.849
				NFS vs. All FS	<0.001 ***	0.936
**CBCL Externalizing**	10.261	3	0.016	NFS vs. All FS	<0.001 ***	0.679
**CBCL Total**	10.658	3	0.014	Color + Presentation FS vs. NFS	0.004 **	0.308
				Texture FS vs. All FS	0.009 **	0.402
				Color + Presentation FS vs. All FS	0.025 *	0.298

Legend: Child Behavior Checklist (CBCL); food selectivity (FS); no food selectivity (NFS); Color + Presentation + Texture FS (All_FS). Note: χ^2^ = chi-square statistic; df = degrees of freedom. *p*-value * = *p* < 0.05, ** = *p* < 0.01, *** = *p* < 0.001.

**Table 7:** Multiple regression analysis statistics of ABAS-II and CBCL subscales.

Model	R^2^	Predictor	B	SE	β	t	*p*
**ABAS_GAC**	0.176	FS_Type	−2.386	0.806	−0.161	−2.961	0.003 **
		Gender	−4.652	2.019	−0.125	−2.304	0.022 *
		Age_months	−0.373	0.076	−0.277	−4.924	<0.001 ***
		IQ	−7.965	1.807	−0.264	−4.409	<0.001 ***
		CSS_ADOS-2	−1.178	0.607	−0.114	−1.941	0.053
**ABAS_DAC**	0.164	FS_Type	−1.311	0.911	−0.079	−1.439	0.151
		Gender	−4.442	2.282	−0.107	−1.947	0.053
		Age_months	−0.379	0.086	−0.251	−4.427	<0.001 ***
		IQ	−9.594	2.042	−0.284	−4.699	<0.001 ***
		CSS_ADOS-2	−1.623	0.686	−0.14	−2.366	0.019 *
**ABAS_DAS**	0.187	FS_Type	−1.407	0.775	−0.098	−1.815	0.071
		Gender	−4.193	1.942	−0.117	−2.159	0.032 *
		Age_months	−0.373	0.073	−0.286	−5.122	<0.001 ***
		IQ	−6.577	1.738	−0.225	−3.785	<0.001 ***
		CSS_ADOS-2	−2.084	0.584	−0.208	−3.571	<0.001 ***
**ABAS_DAP**	0.143	FS_Type	−2.701	0.872	−0.172	−3.099	0.002 **
		Gender	−3.138	2.184	−0.08	−1.437	0.152
		Age_months	−0.387	0.082	−0.271	−4.733	<0.001 ***
		IQ	−6.148	1.954	−0.192	−3.146	0.002 **
		CSS_ADOS-2	−1.308	0.656	−0.119	−1.992	0.047 *
**CBCL_Internalizing**	0.126	FS_Type	3.101	0.567	0.307	5.467	<0.001 ***
		Gender	−0.863	1.421	−0.034	−0.608	0.544
		Age_months	0.059	0.053	0.064	1.113	0.267
		IQ	1.402	1.272	0.068	1.102	0.271
		CSS_ADOS-2	0.882	0.427	0.125	2.066	0.040 *
**CBCL_Externalizing**	0.065	FS_Type	2.203	0.525	0.243	4.197	<0.001 ***
		Gender	−0.589	1.315	−0.026	−0.448	0.654
		Age_months	−0.024	0.049	−0.029	−0.487	0.627
		IQ	0.512	1.177	0.028	0.436	0.664
		CSS_ADOS-2	0.216	0.395	0.034	0.547	0.584
**CBCL_Total**	0.100	FS_Type	2.876	0.56	0.292	5.137	<0.001 ***
		Gender	−0.346	1.403	−0.014	−0.246	0.805
		Age_months	0.019	0.053	0.022	0.367	0.714
		IQ	0.603	1.255	0.03	0.481	0.631
		CSS_ADOS-2	0.636	0.422	0.092	1.509	0.132

Legend: Child Behavior Checklist (CBCL); food selectivity type (FS_Type); Autism Diagnostic Observation Schedule Second Edition (ADOS-2); calibrated severity score (CSS); Adaptive Behavior Assessment System-second edition (ABAS); General Adaptive Composite (GAC); conceptual adaptive domain (DAC); social adaptive domain (DAS). Note: B = unstandardized coefficient; SE = standard error; β = standardized coefficient; t = t-statistic; *p* = significance value; R^2^ = variance explained by the model. *p*-value * = *p* < 0.05, ** = *p* < 0.01, *** = *p* < 0.001.

## Data Availability

Data supporting the findings of this study can be made available on request.
